# Socioeconomic differences in smoking in Jordan, Lebanon, Syria, and Palestine: A cross-sectional analysis of national surveys

**DOI:** 10.1371/journal.pone.0189829

**Published:** 2018-01-30

**Authors:** Sawsan Abdulrahim, Mohammed Jawad

**Affiliations:** 1 Department of Health Promotion and Community Health, Faculty of Health Sciences, American University of Beirut, Beirut, Lebanon; 2 Public Health Policy Evaluation Unit, Imperial College London, Hammersmith, United Kingdom; University of Washington, UNITED STATES

## Abstract

**Introduction:**

The association between education and wealth, as fundamental determinants of health, and smoking is well-established. Yet, social inequalities have received little attention in the expanding field of tobacco research in the Arab region. In this study, we examine inequalities in cigarette smoking by education and wealth in four Arab countries.

**Methods:**

Utilizing the most recently available population-level data sets (Syria 2009 PAPFAM, Jordan 2012 DHS, Palestine 2010 Family Health Survey, and Lebanon 2004 PAPFAM), we tested the association between cigarette smoking and education and wealth–controlling for age, marital status, and region of residence–for each country, and among men and women depending on data availability.

**Results:**

Cigarette smoking prevalence among Arab men is high– 51.3% in Syria, 39.7% in Palestine, and 42.1% in Lebanon; among women, prevalence is 8.4% in Syria, 10.9% in Jordan, and 24.3% Lebanon. Cigarette smoking shows the expected patterns inequalities by education among men in Syria, Palestine, and Lebanon, and among women in Jordan and Lebanon. On the other hand, wealth does not show a clear pattern in its association with cigarette smoking and, in some cases (men in Palestine and women in Syria) the behavioral risk is higher among the wealthiest.

**Conclusions:**

Available data from 2004–2012 show that cigarette smoking among men and women in the four Arab countries is predominant among those with limited access to education as a fundamental cause. The weak or absent negative association between wealth and cigarette smoking suggests that access to material resources does not precipitate a reduction in the consumption of tobacco.

## Introduction

Ample evidence has shown that tobacco control efforts in high-income countries resulted in social inequalities in cigarette smoking and a concentration of this negative health behavior among the disadvantaged. Since the 1960s, data from the United States and northern Europe have consistently shown that persons with low socioeconomic status (SES) report higher smoking rates and lower quit rates than those with high SES [1–4]. This has been shown for both men and women. The negative SES-smoking association persists irrespective of the measure of SES used, although it is stronger for education than income. These trends suggest that public health interventions focused solely on reducing cigarette smoking, at the expense of addressing structural determinants of negative health behaviors, have been effective with advantaged social groups but ineffective in reaching those with low education and low income [2, 5–7].

Social inequalities in cigarette smoking have also been shown in China and India, which have two of the highest smoking prevalence rates worldwide, particularly when education is examined as the SES measure. In both countries, high education groups are less likely to smoke and more likely to quit than those with low education [8–10]. The negative social gradient pattern is also present among urban youth in India, with one study showing that, despite the relatively high cost of cigarettes, public school students (a proxy measure of low SES) smoke cigarettes at higher rates compared to those who attend private schools [11].

Studies on social inequalities in cigarette smoking in low and middle income countries (LMICs) have shown mixed results. In Bangladesh and Ghana, cigarette smoking shows an inverse association with SES irrespective of whether it is measured through education or wealth [12, 13]. Higher education and higher wealth are also associated with lower levels of smoking among men in Colombia, a country considered to be in a late stage of the tobacco epidemic whereby the rates of smoking among men have been decreasing since the late 1990s [14]. In Madagascar, on the other hand, analysis of the 2008–2009 Demographic and Health Survey (DHS) data revealed no association between smoking and SES among men [15]. This absence of an association was explained by the fact that Madagascar is still in an early stage of the tobacco epidemic and has only recently supported tobacco control policies.

Burgeoning evidence on social inequalities in cigarette smoking in many LMICs around the world has raised questions about how public health systems in these countries should respond to one of the most important behavioral risk factors of non-communicable diseases (NCDs). Arab countries of the Eastern Mediterranean are no exception. Recent publications on NCDs in the Arab region have paid special attention to the persistently high rates of cigarette smoking and proposed the implementation of control efforts that have been tested in high-income countries [16] [17]. Since the 1980s, most Arab countries have shown very slow declines in cigarette smoking among men, and some countries (Jordan, Lebanon, and Syria) exhibit relatively high rates of cigarette smoking among women [18]. Further, the increase in waterpipe tobacco smoking in the region, particularly among youth, poses serious concerns for the prospects of health in the coming decades [19, 20].

Social inequalities have received little if any attention in the expanding field of tobacco research in the Arab region. Most writings continue to call for control measures that focus on awareness campaigns and policies that target individual behavior. Following global trends, as tobacco control measures succeed in reducing overall smoking prevalence, the behavior is expected to become increasingly concentrated in low SES groups in Arab countries. In a region where public health systems are under-funded and ill-equipped to address the disproportionate burden of smoking-related NCDs, robust evidence on social inequalities in this important behavioral risk factor among Arab populations is scarce. Only a handful of studies have examined associations between SES and cigarette smoking, revealing that the behavior is inversely associated with both education among adults [21, 22] and parental education among adolescents [23–26].

In this study, we analyze the most recently available population-level data in Syria, Jordan, Palestine, and Lebanon to draw a baseline of the patterning of cigarette smoking by education and wealth. We hypothesize that, for both men and women in the four countries, cigarette smoking displays a negative SES pattern, whereby those with low education and low wealth will smoke at higher rates compared to those with high education and wealth. The present baseline analysis is intended to advocate for tobacco control efforts that take social inequalities into account, and to provide guiding evidence to health systems as they prepare to address the disproportionate impact of smoking-related NCDs on disadvantaged social groups.

## Materials and methods

### Sample and data

We utilized the most recently available population-level surveys on Syria, Jordan, Palestine, and Lebanon that include data on smoking and two SES measures (education and wealth). Given this study is based on an analysis of publically available, anonymized data, it was considered exempt from an Institutional Review Board approval.

For Syria and Lebanon, we used survey data gathered by the Pan Arab Project for Family Health (PAPFAM). PAPFAM gathers nationally representative household data on family health by employing a stratified, multi-stage, probability sampling design [27]. The most recent PAPFAM surveys in Syria (2009) and Lebanon (2004) include data on cigarette smoking for both men and women. For Palestine, we used the 2010 Palestinian Family Survey carried out by the Palestinian Central Bureau of Statistics based on the standard methodology of UNICEF’s Multiple Indicator Cluster Survey (MICS) [28]. Although MICS includes data on cigarette smoking for both men and women, we limited the analysis in this study to men only as smoking prevalence among Palestinian women was very low (1.2%), resulting in small cell counts on bivariate analyses by SES.

For Jordan, we analyzed the most recent Demographic and Health Survey (DHS) collected in the country, DHS 2012. The DHS collects nationally representative data on the health and nutrition of women and children in LMICs by employing a stratified, two-stage, geographically clustered, probability sample design [29]. As the variable on cigarette smoking in the Jordan DHS is in the women’s data file, our findings are limited to ever-married women of reproductive age only.

In all four data sets, we restricted our analysis to the young and middle adulthood age bracket (20–49 years of age). The final sample sizes are: Syria men = 24,615; Syria women = 24,666; Jordan women = 11,113; Palestine men = 14,739; Lebanon men = 4,982; Lebanon women = 5,178.

### Measures

Our outcome measure is current cigarette smoking, ascertained from the Syria and Lebanon PAPFAM surveys as “What is your smoking status?” and which we categorized as “current smoker” versus “past/never-smoker.” In the Palestinian Family Survey, smoking status was assessed through the following question: “Does [named person in household] smoke?” (Options: yes, cigarettes; yes, pipe; yes, narghile; ex-smoker; does not smoke; and never smoked). We dichotomized the response options into “yes, cigarettes” versus all other categories. In the Jordan DHS, smoking status was assessed through the following question: “Do you currently smoke cigarettes?” (Options: yes/no).

Independent variables are education and wealth/income. Education was standardized across the four data sets and grouped into four categories: Less than primary (including illiterate and can read/write); primary (6^th^ grade); preparatory (9^th^ grade); and secondary or higher (12^th^ grade and university education or higher), except in Jordan where the categories were no education, completed primary, completed secondary, and completed higher than secondary. In Syria, Jordan, and Palestine, we used the wealth index as a measure of household economic standing. The wealth index was calculated after conducting principal components analyses on a list of questions relating to household assets and dividing them into quintiles [30]. In Lebanon, where the PAPFAM survey collected data on income but not assets, we categorized income into tertiles (Less than $500/month–low; $500–1,000/month–middle; and greater than $1,000/month–high).

In all multivariate analysis, we adjusted for the following confounders: age, marital status, and area of residence (urban, rural, or refugee camp) or region (Lebanon). Age was standardized across all datasets and coded as 20–29, 30–39 and 40–49 years. Marital status in Syria, Palestine, and Lebanon was coded as “single” versus “ever-married” (including divorced and widowed). In Jordan, we did not adjust for marital status as the DHS included ever-married women only. Area of residence was coded as “urban” versus “rural” in Syria and Jordan, and “urban,” “rural,” or “refugee camp” in Palestine. In the Lebanon PAPFAM, where no data on urban or rural residence existed, the country was divided into five geographical areas: Beirut, Mount Lebanon, Bekaa (Eastern Governorate), North, and South [31].

### Statistical analysis

For each survey, we calculated the prevalence of cigarette smoking across all independent variables in each country; this is presented for men and women in Syria and Lebanon; women only in Jordan; and men only in Palestine. Following, we ran forced, adjusted multivariable logistic regression models to test the association between cigarette smoking and education and wealth, adjusting for age, marital status, and area of residence/region. We report adjusted odds ratios (AORs) with 95% confidence intervals (95% CIs). To assess for multicollinearity between independent variables, we calculated the variance inflation factor (VIF) for each logistic regression model. All VIFs were below five, indicating a reasonable assumption of independence between variables. Sampling weights were used to account for the complex, multi-stage design of all surveys. We performed all statistical analyses using SPSS Version 23.

## Results

### Prevalence of cigarette smoking

Tables 1 and 2 present the prevalence of cigarette smoking by sociodemographic characteristics for men and women. Among men, overall cigarette smoking prevalence is 51.3% in Syria, 39.7% in Palestine, and 42.1% Lebanon. Smoking increases with increasing age and is much higher among ever-married than single men; it is higher among urban men in Syria, whereas, in Palestine, it is higher among rural men compared to those who live in urban areas or refugee camps. As expected, smoking prevalence generally decreases with increasing education (although, in Syria and Palestine, it increases slightly at the primary education level before it begins to drop again) and is lowest among men with secondary education or higher. Except for Lebanon, the association between wealth and smoking is less clear. In Syria and Palestine, only the richest men smoke at a slightly lower rate compared to those in all other wealth categories. In Lebanon, on the other hand, smoking prevalence shows a clear negative association with income.

**Table 1:** Prevalence of current cigarette smoking among men in Syria, Palestine, and Lebanon by sociodemographic characteristics.

		Syria Men		Palestine Men		Lebanon Men	
		N	Prevalence n (%)	N	Prevalence n (%)	N	Prevalence n (%)
**Age**	20–29	11295	5131 (45.4)	6585	2202 (33.4)	2135	664 (31.1)
	30–39	7440	4187 (56.3)	4545	2013 (44.3)	1546	722 (46.7)
	40–49	5880	3319 (56.4)	3602	1633 (45.3)	1303	714 (54.8)
**Marital Status**	Single	10168	4356 (42.8)	5312	1703 (32.1)	2636	868 (32.9)
	Ever-married	14447	8281 (57.3)	9420	4145 (44.0)	2336	1228 (52.6)
**Education**	Secondary or greater	7246	2767 (38.2)	6761	2060 (30.5)	2074	645 (31.1)
	Preparatory	4027	2231 (55.4)	4529	2087 (46.1)	1256	592 (47.1)
	Primary	9457	5576 (59.0)	2440	1254 (51.4)	1214	625 (51.5)
	Less than Primary	3877	2061 (53.2)	983	441 (44.9)	423	236 (55.8)
**Wealth**	Richest	4611	2095 (45.4)	2968	1071 (36.1)	-	-
	Fourth	5066	2573 (50.8)	3091	1261 (40.8)	557	179 (32.1)
	Middle	4988	2664 (53.4)	3022	1204 (38.8)	2289	910 (39.8)
	Second	4995	2674 (53.5)	2939	1231 (41.9)	1931	921 (47.7)
	Poorest	4955	2631 (53.1)	2721	1081 (39.9)	-	-
**Area of Residence**	Urban	12388	6471 (52.2)	10485	4036 (38.5)	-	-
	Rural	12227	6166 (50.4)	2759	1193 (43.2)	-	-
	Refugee Camp			1488	619 (41.6)	-	-
**Region (Lebanon)**	Beirut					476	189 (39.7)
	Mt Lebanon					2028	874 (43.1)
	Bekaa					610	259 (42.5)
	North					1069	444 (41.5)
	South					799	333 (41.7)

**Table 2:** Prevalence of current cigarette smoking among women in Syria, Jordan, and Lebanon, by sociodemographic characteristics.

		Syria Women		Jordan Women		Lebanon Women	
		N	Prevalence n (%)	N	Prevalence n (%)	N	Prevalence n (%)
**Age**	20–29	10949	490 (4.5)	3300	212 (8.0)	1885	222 (11.8)
	30–39	7909	736 (9.3)	4333	340 (9.4)	1746	480 (27.5)
	40–49	5808	838 (14.4)	3480	417 (15.1)	1547	557 (36.0)
**Marital Status**	Single	7079	382 (5.4)	-	-	2087	269 (12.9)
	Ever-married	17586	1682 (9.6)	-	-	3087	989 (32.0)
**Education**	Secondary or greater	6459	471 (7.3)	-	-	2344	380 (16.2)
	Preparatory	3357	344 (10.2)	-	-	1274	346 (27.2)
	Primary	8134	642 (7.9)	-	-	1027	365 (35.5)
	Less than Primary	6708	605 (9.0)	-	-	506	164 (32.4)
**Education (Jordan)**	More than secondary	-	-	3547	188 (7.4)	-	-
	Secondary	-	-	6197	595 (11.8)	-	-
	Primary	-	-	962	134 (16.1)	-	-
	None	-	-	407	52 (17.2)	-	-
**Wealth**	Richest	4838	506 (10.5)	1102	149 (15.5)	-	-
	Fourth	4826	414 (8.6)	2020	161 (8.8)	578	88 (15.2)
	Middle	4852	425 (8.8)	2547	199 (8.9)	2302	514 (22.3)
	Second	4959	327 (6.6)	2824	216 (10.9)	2070	590 (28.5)
	Poorest	5191	392 (7.6)	2620	244 (10.9)	-	-
**Area of Residence**	Urban	12354	1192 (9.6)	3267	788 (11.8)	-	-
	Rural	12312	872 (7.1)	7846	181 (6.4)	-	-
**Region (Lebanon)**	Beirut	-	-	-	-	574	191 (33.3)
	Mt Lebanon	-	-	-	-	2153	520 (24.2)
	Bekaa	-	-	-	-	623	116 (18.6)
	North	-	-	-	-	963	242 (25.1)
	South	-	-	-	-	866	190 (21.9)

Among women (Table 2), overall cigarette smoking prevalence is 8.4% in Syria, 10.9% in Jordan, and 24.3% Lebanon. Similar to men, smoking prevalence among women increases with increasing age, is much higher among ever-married compared to single women (except in Jordan where the sample includes ever-married women only), and is higher among urban compared to rural residents. In Syria whereby the overall smoking prevalence among women is relatively low (less than 10%), the results do not show a negative association between SES and smoking; indeed, the richest women in Syria exhibit the highest smoking prevalence. Jordan displays a mixed picture in that smoking rates are highest among the richest (15.5%) and the least educated (17.2%). In Lebanon, on the other hand, women who have secondary education or higher and those who fall into the rich wealth category exhibit the lowest smoking prevalence (16.2% and 15.2%, respectively).

Results of adjusted multivariate analysis confirm bivariate trends for both men and women (Tables 3 and 4, respectively). Among men in Syria, Palestine, and Lebanon, education is a predictor of cigarette smoking, with men who have preparatory, primary, and less than primary education exhibiting significantly higher odds of cigarettes smoking compared to those with secondary education or higher. In Syria and Palestine, the highest odds of smoking are among men with primary education (Syria AOR = 2.17, 95% CI = 2.03–2.32; Palestine AOR = 2.25, 95% CI = 2.04–2.49), compared to men with secondary education or higher. In Lebanon, the odds of smoking exhibit a negative gradient; compared to men with secondary education or higher, the AORs increase in a step-wise manner, although with overlapping confidence intervals, with decreasing education: preparatory AOR = 1.87, 95% CI = 1.60–2.18; primary AOR = 1.99, 95% CI = 1.69–2.34; less than primary AOR = 2.33, 95% CI = 1.85–2.94.

**Table 3:** Education and wealth correlates of current cigarette smoking, men.

		Syria Men		Palestine Men		Lebanon Men	
		OR	95% CI	OR	95% CI	OR	95% CI
**Education**	Secondary or greater	1.00	-	1.00	-	1.00	-
	Preparatory	**1.90**	**1.76–2.06**	**1.93**	**1.78–2.09**	**1.87**	**1.60–2.18**
	Primary	**2.17**	**2.03–2.32**	**2.25**	**2.04–2.49**	**1.99**	**1.69–2.34**
	Less than Primary	**1.80**	**1.65–1.96**	**1.77**	**1.54–2.04**	**2.33**	**1.85–2.94**
**Wealth**	Richest	1.00	-	1.00	-	-	-
	Rich	**1.09**	**1.01–1.19**	1.07	0.96–1.19	1.00	-
	Middle	**1.13**	**1.03–1.23**	0.93	0.84–1.04	1.09	0.88–1.34
	Poor	1.09	1.00–1.20	0.97	0.87–1.08	**1.26**	**1.01–1.57**
	Poorest	1.02	0.92–1.12	**0.83**	**0.74–0.94**	-	-

**Note**: Models are adjusted for age, marital status, urban/rural residence in Syria and Palestine (including refugee camp), and region of residence in Lebanon (Beirut versus four other regions)

**Table 4:** Education and wealth correlates of current cigarette smoking, women.

		Syria Women		Jordan Women		Lebanon Women	
		OR	95% CI	OR	95% CI	OR	95% CI
**Education**	Secondary or greater	1.00	-	-	-	1.00	-
	Preparatory	**1.38**	**1.18–1.60**	-	-	**1.54**	**1.28–1.85**
	Primary	1.07	0.94–1.23	-	-	**2.09**	**1.72–2.54**
	Less than Primary	1.15	0.99–1.33	-	-	**1.61**	**1.26–2.05**
**Education (Jordan)**	More than secondary	-	-	1.00	-	-	-
	Secondary	-	-	**1.90**	**1.45–2.49**	-	-
	Primary	-	-	**2.78**	**1.88–4.11**	-	-
	None	-	-	**2.93**	**1.47–5.84**	-	-
**Wealth**	Richest	1.00	-	1.00	-	-	-
	Rich	**0.85**	**0.74–0.98**	**0.52**	**0.37–0.72**	1.00	-
	Middle	0.91	0.79–1.06	**0.50**	**0.35–0.72**	**1.43**	**1.10–1.86**
	Poor	**0.72**	**0.61–0.85**	**0.56**	**0.38–0.82**	**1.84**	**1.40–2.42**
	Poorest	0.91	0.77–1.08	**0.49**	**0.33–0.74**	-	-

**Note**: Models are adjusted for age, marital status, urban/rural residence in Syria and Palestine (including refugee camp), and region of residence in Lebanon (Beirut versus four other regions)

Wealth does not show a clear pattern in its association with cigarette smoking among men and, even in cases where wealth showed an association, this association remained weak. In Syria, compared to men in the richest wealth category, only men in the rich and middle categories exhibit slightly higher odds of smoking (AOR = 1.09, 95% CI = 1.01–1.19 and AOR = 1.13, 95% CI = 1.03–1.23, respectively). Among Palestinian men, wealth does not predict the odds of cigarette smoking; interestingly, being in the poorest category is protective (AOR = 0.83, 95% CI = 0.74–0.94). In Lebanon, men in the poor wealth category have 1.26 times the risk of smoking but this higher risk is only borderline significant.

Among Syrian women, adjusted associations between the two SES measures (education and wealth) and cigarette smoking are generally non-significant. Only women with preparatory education are more likely to smoke compared to those with secondary education or higher (AOR = 1.38, 95% CI = 1.18–1.60). In Jordan, both education and wealth are predictors of cigarette smoking; the least educated had over twice the odds of cigarette use compared with the most educated (AOR 2.93, 95% CI 1.47–5.84). In contrast, the poorest women had around half the odds of cigarette use compared with the richest (AOR 0.49, 95% CI 0.33–0.74). In Lebanon, where women’s cigarette smoking prevalence is two and a half times higher than it is in Syria and Jordan, both education and wealth are strongly predictive. Lebanese women with preparatory, primary, and less than primary education exhibit significantly higher odds of cigarette smoking compared to women with secondary education or higher; in particular, the AOR for preparatory is two times higher (AOR = 2.09, 95% CI = 1.72–2.54). Wealth is also predictive of cigarette smoking among Lebanese women and, despite overlapping confidence intervals, the association shows a gradient–middle AOR = 1.43, 95% CI = 1.10–1.86 and poor AOR = 1.84, 95% CI = 1.40–2.42.

## Discussion

Most Arab countries have been experiencing an epidemiologic transition from communicable to non-communicable diseases as major causes of morbidity and mortality. With tobacco-use being an established risk factor for cardiovascular disease and cancer, reducing its prevalence has thus been advocated by public health researchers and policy makers as one of the “best buys” to reduce the burden of NCDs in the region [16, 17]. Our study sought to address an evident gap in the published literature on the region that has focused on cigarette smoking as an individual health behavior and neglected to examine it through a social inequalities lens. The findings show expected patterns of cigarette smoking inequalities by education, but not by wealth. They also show that men and women display different patterns by education. As hypothesized, those with less than secondary education have an increased risk of cigarette smoking among men in Syria, Palestine, and Lebanon. It is protective among Jordanian and Lebanese women as well, but not among Syrian women. Wealth, on the other hand, does not show a clear pattern and exerts a much weaker protective effect on cigarette smoking among men in Syria and Lebanon, and among Lebanese women only. Strangely, men in the poorest wealth category in Palestine and women in the poor category in Syria have a lower risk of smoking than the richest.

The almost consistent negative association between cigarette smoking and education in the four Arab countries (mostly men but also women in Jordan and Lebanon) suggests that cigarette smoking has declined in the 1990s or early 2000s among individuals who have access to at least secondary education. Our findings can be explained by the diffusion of innovation theory by Rogers in the 1970s [4] and the fundamental cause theory by Link and Phelan [32]. According to Rogers [4], new health ideas and practices are adopted first by socially advantaged individuals and groups, and are taken up relatively late in the diffusion process by the disadvantaged [33]. Link and Phelan [32] utilize a structural framework to explain how education embodies access to knowledge and resources, and therefore enables individuals who possess it to respond to interventions that focus on behavioral change. We conjecture that as scientific evidence on the negative health effects of smoking became widely available in Syria, Jordan, Palestine, and Lebanon, educated individuals in these countries responded to this knowledge by quitting smoking or not starting the habit to begin with. The outcome of this change was that cigarette smoking became concentrated among individuals in these four countries who do not possess the knowledge and resources that education, as a fundamental cause, confers.

The weak or absent negative association between wealth and cigarette smoking, despite the strong negative association with education, is not surprising. Social epidemiological research has provided evidence that education and income tap into different underlying measures of socioeconomic position and associate with different health outcomes differently [34]. Whilst education operates through enhancing health knowledge to reduce cigarette smoking, wealth does not necessarily reduce the consumption of tobacco products. Studies in Egypt have shown a similar pattern with respect to other NCD risk factors; in two studies, education was shown to protect against obesity and diabetes whereas wealth was shown to increase obesity because it associates with greater consumption of high energy foods [35, 36].

As to the absence of a negative association between wealth and cigarette smoking in Palestine, we conjecture that chronic political conflict flattens out the wealth gradient whereby Palestinian men, irrespective of level of wealth, continue to smoke as a coping mechanism. Moreover, the presence of a negative association between wealth and cigarette smoking among Lebanese women in comparison to other patterns in Syria and Jordan highlights that gendered social class representations interact with cultural norms in determining the patterns of female smoking in each country. The relatively low female smoking prevalence in Syria and Jordan may reflect cultural differentiation by social class whereby cigarette smoking for women remains acceptable only among “westernized” social groups who tend to possess more wealth. In Lebanon, on the other hand, where tobacco use by women enjoys cultural acceptability, Lebanese women of all social classes and even rural women smoke at a relatively high rate. In this context of cultural permissiveness, smoking becomes differentiated by social class in the expected direction; women who possess education and wealth begin to shun the habit while women with less access to knowledge and resources continue to smoke.

Our study has several limitations. Firstly, our samples, though nationally representative, were not uniformly comparable in terms of their characteristics. For example, our Jordanian sample was among ever-married women only and there may be important patterns of cigarette use among single women and men in Jordan, which remain unknown to us. However, nationally representative cigarette smoking data from the region are somewhat lacking in open access repositories, so our approach could be considered a reasonable alternative. Secondly, our measures for cigarette prevalence differ slightly between all four countries, which may introduce a measurement bias, though it is unclear the extent to which this would occur or the direction of the bias. Thirdly, we omitted information on other forms of tobacco use, such as waterpipe tobacco because not all surveys capture these. However, waterpipe tobacco smoking, a relatively new phenomenon, is commonly associated with high SES [37], and we expect this pattern to be maintained here in line with the diffusion of innovation model. Finally, our cross-sectional design does not allow us to measure changes in the social patterning of cigarette use over time, and this would be a reasonable next step to gain further understanding of the issue.

To conclude, cigarette smoking among men and women in the four Arab countries is predominant among those with limited access to education as a fundamental cause. The weak or absent negative association between wealth and cigarette smoking suggests that access to material resources does not precipitate a reduction in the consumption of tobacco. One discernable reason for this is the cheap price of cigarettes in the region; to promote good governance in tobacco control, increased taxation on cigarettes is warranted which is likely to act both through prevention and cessation tobacco pathways.
